# Sensitisation of low-threshold bladder afferent fibres to physiological distension following intravesical mitomycin C in mice

**DOI:** 10.1007/s00345-026-06879-z

**Published:** 2026-11-04

**Authors:** Kylie A. Mills, Donna J. Sellers, Russ Chess-Williams, Catherine McDermott

**Affiliations:** Centre for Urology Research, Faculty of Health Sciences and Medicine, Bond University, Robina, Robina, QLD 4229 Australia

**Keywords:** Mitomycin C, Bladder, Sensory nerves, Intravesical

## Abstract

**Purpose:**

Intravesical mitomycin C (MMC) is commonly associated with urinary bladder dysfunction in patients. This study investigated the effect of intravesical MMC on bladder sensory nerve activity during bladder filling in an in vitro murine model.

**Methods:**

Anaesthetised mice were treated intravesically with saline or MMC (1 mg/mL) for 60 min and 24 h later, bladders were isolated and set up for recording of afferent nerve activity during bladder filling and treatment with pharmacological agents.

**Results:**

MMC treatment did not affect bladder compliance but enhanced overall afferent nerve activity throughout bladder filling. This enhancement was observed only in afferent fibres with a low threshold and wide dynamic range; those with a narrow dynamic range were not affected by MMC pretreatment, whilst activity in the minor population of high threshold fibres was depressed after MMC. Intravesical pressure responses to carbachol (1µM) or ATP (10µM) were not affected by MMC pretreatment. Capsaicin (100µM) depressed the rise in nerve activity during bladder filling in control experiments (50% depression), and this effect was reduced (only 20% depression) after MMC treatment (*P* < 0.05). This effect of MMC was observed at both physiological pressures (up to 15mmHg) and at high pressures (40mmHg).

**Conclusions:**

In conclusion, intravesical treatment with MMC results in an enhancement of overall sensory nerve responses to bladder filling, and this was due to a selective sensitization of low threshold, wide dynamic range, afferent fibres. Over the physiological range of pressures, afferent nerve activity was enhanced, which may explain the urinary urgency and frequency encounter by patients after MMC treatment.

## Introduction

Mitomycin C (MMC) is an alkylating agent often used in the treatment of bladder cancer, where it is administered by intravesical delivery directly into the bladder lumen, typically following transurethral resection of the bladder tumour. Intravesical chemotherapy facilitates the delivery of high drug concentrations, while minimising the risk of systemic side effects. However, intravesical MMC is known to cause local urological side effects in up to 41% of patients, which range from mild irritative symptoms to severe debilitating pain, which can limit the tolerability of treatment [12, 13, 20]. Intravesical MMC causes aseptic cystitis, with the most frequently reported urological side effects including increased urinary frequency and urgency, and in some cases, painful urination. A range of treatments are prescribed for the management of local side effects, including antihistamines, anticholinergics, non-steroidal anti-inflammatory drugs, steroids and alpha-blockers [6]. Luckenbaugh et al. [13] developed the first treatment algorithm for the management of MMC-induced cystitis, with an escalating approach based on the severity of symptomology.

Studies investigating intravesical MMC treatment in rodent models have provided some mechanistic insight into the local effects of the treatment. Intravesical MMC was associated with chronic inflammation, fibrosis and impaired wound healing in the bladder of treated rats [10], and a persistent decrease in bladder volume and compliance that was still evident 3 weeks following treatment [14]. West et al. [22] reported increased urinary frequency in mice following a single intravesical dose of MMC, which was associated with increased frequency of spontaneous phasic contractile activity. These voiding and functional changes were ameliorated by ibuprofen treatment, further supporting the role of inflammation in the development of voiding symptoms. MMC pre-treatment results in transient changes in urothelial signalling in human urothelial cultures, while raised inflammatory cytokines and nitric oxide release persisted 7-day following treatment [11]. Pathological bladder conditions resulting from inflammation or injury can alter sensory signalling, with changes in the sensitivity of bladder afferents linked to storage and voiding symptoms, as well as pain [7, 15, 25]. The impact of intravesical chemotherapy on bladder sensory nerve function is currently unknown.

Intravesical MMC is associated with lower urinary tract symptoms, including urgency, frequency, painful urination and pelvic pain in some bladder cancer patients [12]. Altered sensory signalling in the bladder is likely to play a role in the development of urological complications in patients following intravesical MMC; however, no studies to date have assessed the impact of intravesical MMC on bladder sensory nerves. Therefore, the aim of this study was to determine if intravesical MMC treatment affects bladder afferent nerve activity in mice.

## Materials and methods

### Animal model

All procedures were performed in accordance with the Australian Code for the Care and Use of Animals for Scientific Purposes and with the approval of the University of Queensland Animal Ethics Committee. Adult female C57BL/6JArc mice (12–14 weeks in age) were used for this study and housed under environmentally controlled conditions, with 12-hour light-dark cycles and access to food and water ad *libitum*. Animals were randomly allocated into two experimental groups: Control or MMC-treated. Mice in the control group weighed 21.6 ± 0.45 g (*n* = 8), while those treated with MMC weighed 22.1 ± 0.48 g (*p* = 0.45) (*n* = 7).

### Intravesical treatment

Mice were anaesthetised with 1–3% isoflurane gas and micturition was induced by mild abdominal massage to prevent dilution of intravesical solutions. Bladder catheterisation was then performed with a sterile intravenous catheter (Terumo, Japan) through the urethra. Thirty µL of solution (saline for control animals or 1 mg/mL MMC, clinical dose) was instilled through the catheter. The catheter was removed, and mice were allowed to run freely in their cage with access to food, but not water, for 1 h on Whatman Filter Paper no. 1, ensuring they did not void during this treatment period. Micturition was induced 1-hour post-intravesical instillation to expel MMC or saline solution.

### Bladder afferent nerve recording and analysis

A bladder afferent nerve recording preparation was used for functional bladder studies as previously described [15]. Mice were euthanised by cervical dislocation 24 h following intravesical treatment. The pelvic region was dissected and placed into a 30mL bath, continually superfused with gassed (95%O_2_/5% CO_2_) Krebs-bicarbonate solution (composition in mM: NaCl 118, NaHCO_3_ 24.9, CaCl_2_ 1.9, MgSO_4_ 1.15, KCl 4.7, KH_2_PO_4_ 1.15, and D-glucose 11.7) at a rate of 6mL/min at a temperature of 37 °C.

The ureters were ligated and a two-way catheter was inserted into the bladder dome to allow measurement of intravesical pressure and bladder emptying. A urethral catheter was inserted to allow ramp bladder distension with isotonic saline at 30 µl/min to a maximum intravesical pressure of 40mmHg.

The bladder afferent nerves were divided into fine branches, and one branch was cut between the bladder and the spinal cord, and the free end was inserted into a suction electrode. A ramp distension to 40mmHg was performed to confirm that the selected branch was responsive to bladder distension. Multi-unit afferent nerve activity was then recorded by a Neurolog headstage (NL100, Digitimer Ltd, UK), amplified (NL014, Digitimer Ltd), filtered (NL125, band pass filter, Digitimer Ltd) and passed through a 50/60Hz electrical noise eliminator (Humbug, Quest Scientific, Canada) to a Micro1401 analogue to digital interface (Cambridge Electronic Design, UK) and then visualised on a computer using Spike2 software (version 7.1, Cambridge Electronic Design, UK).

After 30 min equilibration, three bladder distensions were performed to ensure consistent pressure and nerve responses. Bladder accommodation was performed by filling at the same rate to 15mmHg and allowing the pressure and nerve activity to plateau. From this plateau the muscle and nerve responses to ATP (1mM) and carbachol (1µM) were also measured.

A further bladder distension to 40mmHg was followed by serosal application of capsaicin (10µM) for 10 min and then an additional application of capsaicin (100µM) to desensitize TRPV1 receptors. After 10 min of exposure, another distension was performed, and the effect of capsaicin on bladder compliance and afferent nerve activity was determined. The reduction in afferent nerve activity was represented as a percentage of the response recorded before capsaicin application.

Multi-unit nerve activity was quantified using Spike2 software which counted the number of action potentials that crossed a pre-set threshold. The afferent nerve response during bladder distension was calculated by measuring the afferent activity (imp/sec) at each intravesical pressure increment up to 40mmHg. Single-unit (single nerve fibre) sorting was performed using the Spike2 wave-mark analysis function, which discriminates between distinct action potentials to identify the individual nerve fibres in each preparation.

### Statistical analysis

All experiments were randomized, with each experimental protocol started on a different day. Results are expressed as mean ± standard error of the mean (SEM). Data were analysed using Graphpad Prism version 9 software (GraphPad, SanDiego, CA). Significance levels were defined as *P* < 0.05 (*). Unpaired Student’s test was used to compare mean nerve activity responses at each pressure point. In addition, the area under the curve (AUC) was calculated over the full pressure range of total nerve activity responses and compared between the control and MMC-treated groups.

## Results

### Effects of intravesical MMC on bladder compliance and mechanosensitive bladder afferents

**Fig. 1:**
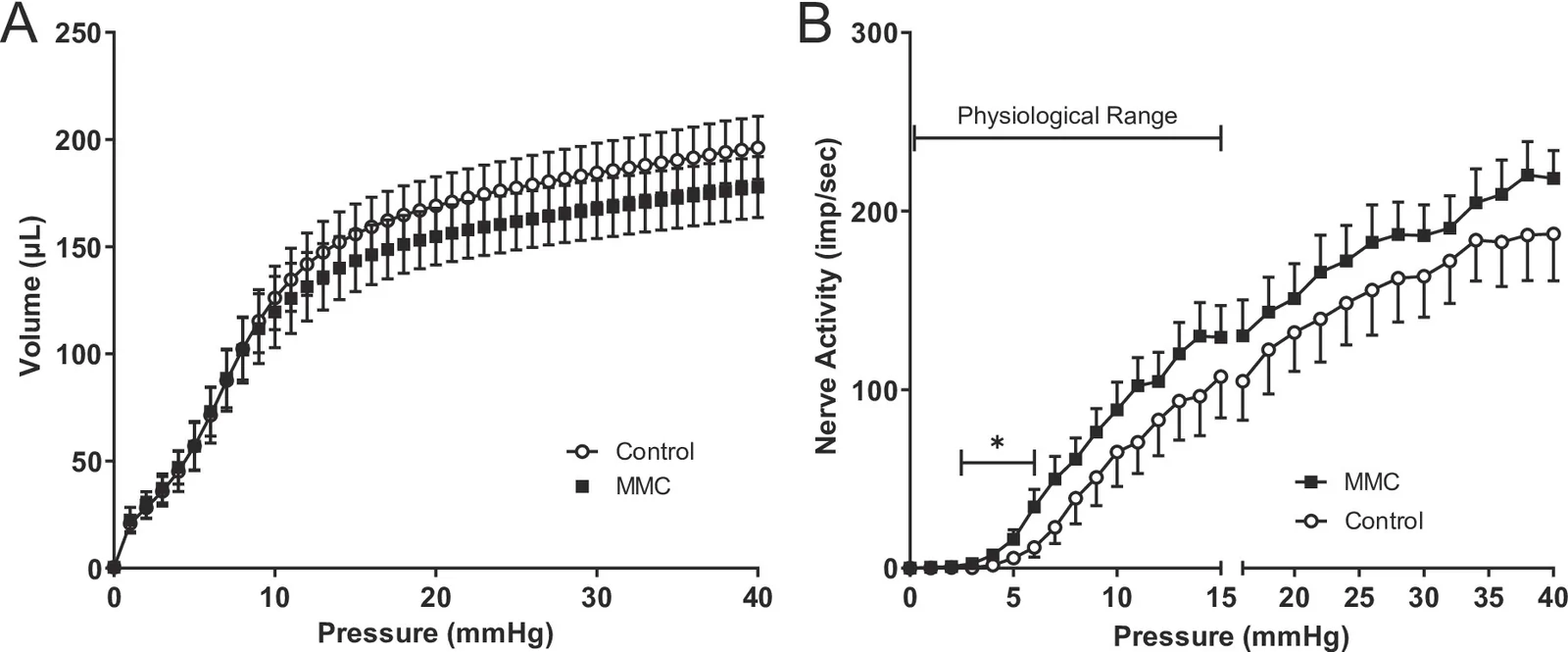
Effect of intravesical MMC on bladder compliance pressure (**A**) and total bladder afferent nerve activity (**B**) during filling to 40 mmHg intravesical pressure. Data represented as mean ± SEM (*n* ≥ 7) and analysed by unpaired Students t test (*p < 0.05 control vs. MMC)

Twenty-four hours following intravesical treatment, bladders were filled at a rate of 30 µL/min and an increase in intravesical pressure and afferent nerve activity was noted (Fig. 1). No significant difference in the volume-pressure relationship of the bladder was observed between control and MMC-treated animals (Fig. 1A), indicating that bladder compliance was unchanged over the physiological (0–15 mmHg) and supraphysiological (> 15 mmHg) pressure ranges tested.

At lower physiological pressures (3–6 mmHg) mean afferent nerve activity was significantly higher in the MMC-treated group compared to controls (Fig. 1B). Comparison of mean area under the curve revealed a significant difference between total nerve activity responses in the MMC-treated and control groups over the full pressure range, with a significant increase evident in the MMC group (Control 4491 ± 352 imp mmHg/s vs. MMC 5363 ± 265 imp mmHg/s, *p* = 0.028).

### Single unit analysis

**Fig. 2:**
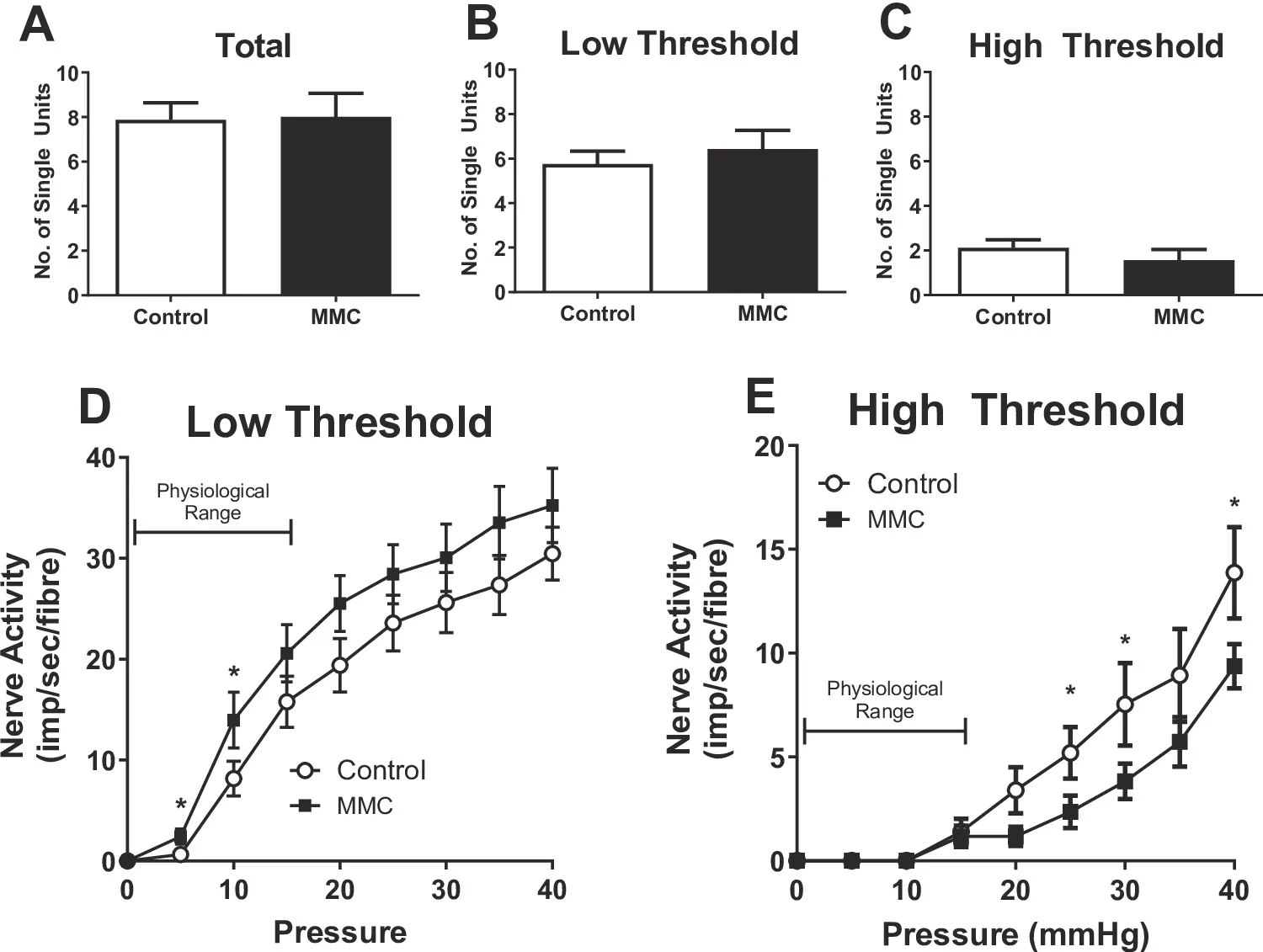
The number of distinct nerve fibres contributing to total nerve activity in each bladder (**A**) and those identified as low (**B**) or high (**C**) threshold nerve fibres in the control and MMC treated group. Nerve activity of low (*n* ≥ 48) (**D**) and high (*n* ≥ 11) (**E**) threshold fibres. Data represented as mean ± SEM and analysed by unpaired Students t test (*p < 0.05 control vs. MMC)

Single fibres within the nerve branch were identified using Spike2 software wavemark analysis. The total number of distinct individual nerve fibres contributing to the total nerve response measured was not significantly different between the groups (Fig. 2A). Each individual fibre was then classified as either low or high threshold, based on the pressure threshold at which it became active. The low threshold fibres become active at low pressures, just after bladder filling begins, while high threshold nerve fibres become active at around 15 mmHg. The number of low and high-threshold nerve fibres was unchanged by MMC treatments (Fig. 2B and C).

**Fig. 3:**
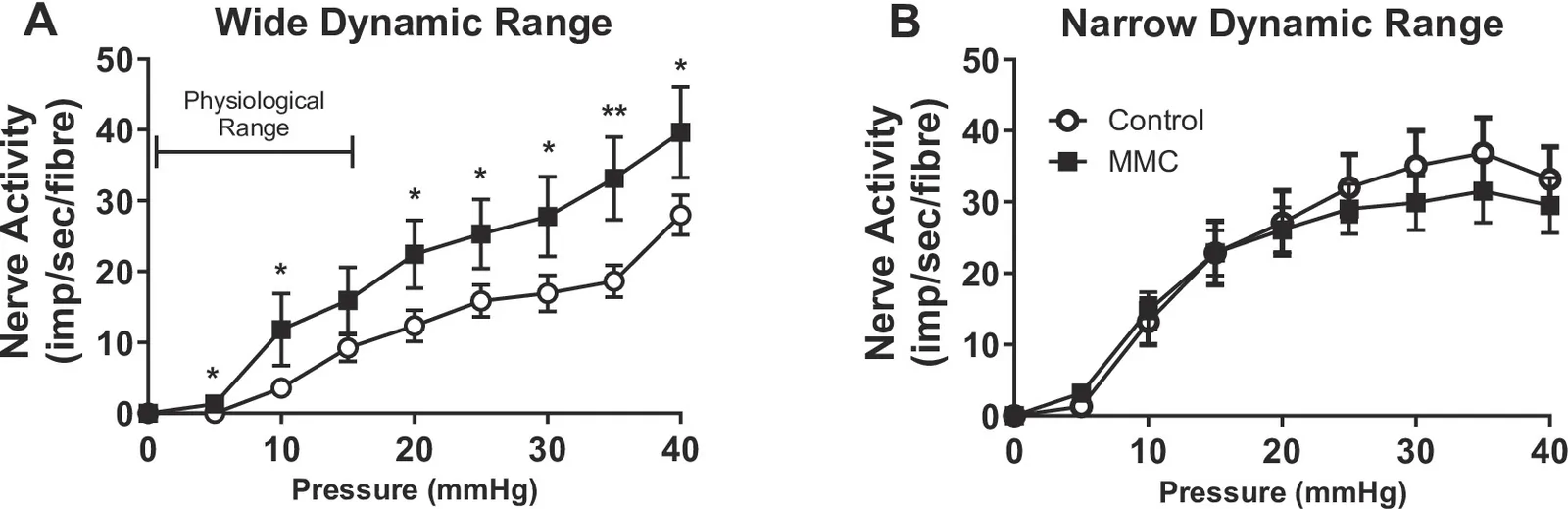
Bladder afferent nerve activity in low threshold (**A**) wide dynamic range fibres and (**B**) narrow dynamic range fibres (*n* ≥ 20) in control and MMC treated mice. Data presented as mean ± SEM and analysed using unpaired Student’s t-test (*p < 0.05 control vs. MMC; **p < 0.01 control vs. MMC)

The low-threshold fibres, which are responsible for normal bladder filling sensations, were significantly more active in the MMC-treated group up to a pressure of 10 mmHg, which is within the physiological range (Fig. 2D). While the activity of the high threshold fibres was significantly reduced in the MMC group at higher pressures (Fig. 2E). Two distinct response patterns were evident within the low-threshold fibres. In the first group, low-threshold afferents showed a gradual increase in excitability up to maximum distension, exhibiting a wide dynamic range (WDR). The second group exhibited a narrow dynamic range (NDR), where activity plateaued or decreased after intravesical pressures exceeded approximately 15 mmHg. The effect of intravesical MMC on low threshold fibres was due solely to enhanced activity in WDR fibres (Fig. 3A) over the physiological and supraphysiological pressure range, while activity in NDR fibres was unchanged by MMC treatment (Fig. 3B).

### Responses to pharmacological agents

**Fig. 4:**
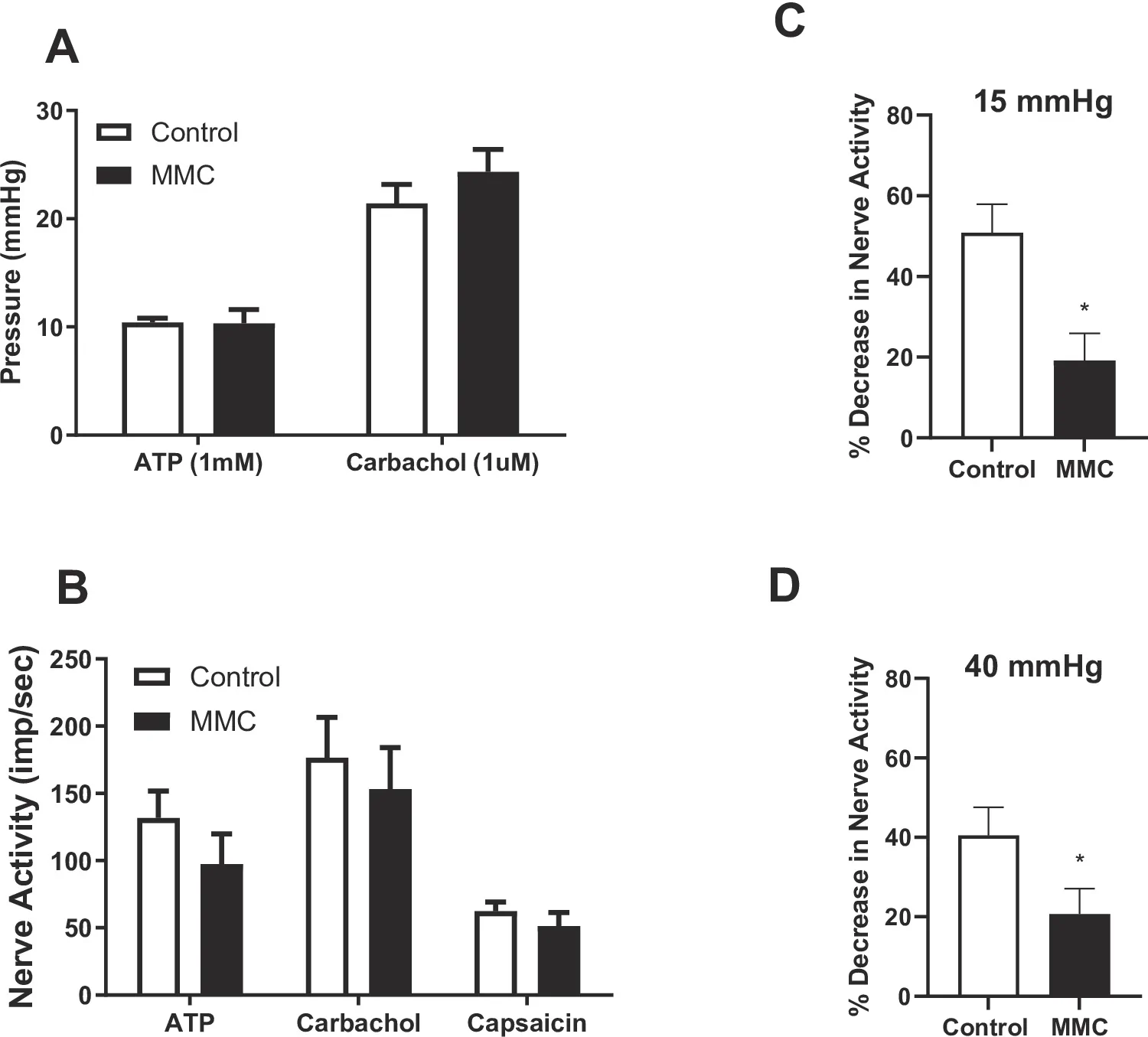
**A** Intravesical pressure and **B** nerve activity responses of bladders from control and MMC-treated mice to ATP (1mM), carbachol (1µM) and capsaicin (100µM). **C** & **D** Percentage reduction in nerve activity following capsaicin. Data represent mean ± SEM and were analysed using an unpaired Student’s t-test

Bladders were filled to 20 mmHg and allowed to equilibrate until pressure and nerve activity were stable before quantifying the responses to various agonists. Extraluminal application of the purinergic agonist ATP and the cholinergic agonist carbachol resulted in significant increases in intravesical pressure and afferent nerve activity (Fig. 4A and B). Neither pressure nor afferent nerve responses to these agents were significantly affected by pretreatment with intravesical MMC.

Capsaicin elicited afferent nerve firing, which was similar between the control and MMC-pretreated groups, but did not alter bladder compliance determined from the volume-pressure relationship of subsequent bladder distension to 40 mmHg (data not shown). The nerve activity response to distension was consistently reduced following capsaicin; however, the percentage decrease was significantly less following MMC pre-treatment (Fig. 4C and D).

## Discussion

In clinical urology, intravesical MMC therapy for non-muscle-invasive bladder cancer (NMIBC) is frequently limited by a high incidence of lower urinary tract symptoms. Patients routinely report debilitating urinary urgency, frequency, and dysuria (chemical cystitis) that peak within 24 to 48 h of instillation. Historically, these symptoms were attributed to generalized chemical inflammation, but the mechanisms involved in causing these symptoms are unknown. Bladder sensory nerves play an important role in normal bladder function, relaying information regarding the degree of bladder fullness via low-threshold Aδ fibres to the central nervous system, while high-threshold C fibres, which are normally silent under physiological conditions, can be activated by chemical irritation, inflammation or noxious stimuli, contributing to pain [26]. This study examined afferent nerve activity in a murine model, where the dose of MMC and the duration of treatment mimicked that used in the clinical situation.

Our previous study has shown this treatment in mice results in changes in micturition with increased urinary frequency 24 h after MMC treatment [22], the time point investigated in the present study. In the isolated bladders, 24 h after MMC treatment, compliance was similar in bladders from control and treated animals. In both groups, overall afferent nerve activity increased as bladders were filled at a physiological rate (30µL/hr), but the level of nerve activity throughout the filling stage was significantly greater in bladders from MMC pretreated animals.

Enhanced afferent activity has been reported in several models of cystitis, both from chemical exposure and infection [15, 19, 23] as well as in a model of stress-induced voiding dysfunction [16], an effect that is associated with altered voiding behaviours. Here, we report hypersensitivity of low-threshold, wide-dynamic-range afferent fibres following intravesical MMC treatment at the clinically relevant dose and duration of treatment. Similar changes have been observed in a murine model following cyclophosphamide or ifosfamide exposure, mimicking the use of these drugs as systemic chemotherapeutics [15]. The increased urinary frequency and urgency experienced by patients following intravesical treatment and the changes in voiding behaviour reported in mice following local MMC treatment [22] may reflect this sensitisation of low-threshold fibres during normal bladder filling, where micturition thresholds are triggered at lower intravesical pressures. By accelerating the firing rate of low-threshold mechanoreceptors at low volumes, this low-pressure afferent bombardment likely triggers a premature micturition reflex, directly driving the clinical presentation of urinary frequency and urgency even in the absence of high detrusor pressures. MMC-pretreatment also affected high threshold afferent fibres, but surprisingly the activity of these fibres was depressed and difficult to interpret. The total number of nerve fibres and the ratio of low and high threshold fibres was similar in control and treated animals, and the number of low-threshold fibres greatly outnumbered the high threshold fibres by more than four-fold in both groups. Thus the contribution of low threshold fibres to overall afferent activity was great throughout the whole filling cycle, and even at the top of the filling cycle, overall afferent activity was increased after MMC.

During bladder filling, urothelial stretch induces the release of mediators that include acetylcholine and ATP, and these can exert effects on the detrusor smooth muscle and afferent nerves. The effects of MMC on the release of these mediators 24 h after treatment has been examined previously in cultured urothelial cells and the basal release of acetylcholine found to be enhanced after MMC [11]. In the present study, the responsiveness of the detrusor and afferent nerves to these mediators was assessed. The muscarinic agonist carbachol and the purinergic agonist ATP increased both intravesical pressure responses and afferent nerve activity. Pressure responses to carbachol are mediated via M3 receptors located on the detrusor smooth muscle [2, 18], whilst the afferent responses to carbachol are more complicated, involving direct effects on afferent nerves, but also nerve activity induced indirectly by the increase in detrusor contraction [4]. The mechanisms involved in the direct effects of carbachol on nerve activity are therefore difficult to assess and this has resulted in reports of both depressed [4] and enhanced [26] afferent activity following muscarinic receptor stimulation. Similarly for responses to ATP, both pressure (via detrusor P2 × 1 receptors) and direct nerve stimulation (predominantly P2 × 3 receptors) contribute to the overall afferent response, but for ATP a direct activation of predominantly P2 × 3 receptors is well established [21]. In the MMC pretreated animals neither the pressure responses nor the afferent responses to ATP were affected, indicating that detrusor and afferent nerve responsiveness are unlikely altered by MMC.

In contrast, the potent TRPV1 receptor agonist capsaicin elicited only an afferent nerve response without any change in intravesical pressure. It has previously been reported that TRPV1 plays an important role in the excitability of low-threshold bladder afferents [3] and that TRPV1 channels influence the micturition response via a mechanism that enhances afferent nerve responses to distension during bladder filling [8]. The current study confirms this finding, with a significant reduction in the response to bladder filling in control animals following capsaicin, a TRPV1 agonist that causes rapid desensitisation of these receptors. This explains the depression of afferent nerve activity during distension in the present study, even within the physiological pressure range. While application of capsaicin elicited equivalent afferent nerve stimulation in MMC-treated and control bladders, suggesting TRPV1 expression was unchanged, we also found that the reduction in afferent firing in response to bladder filling was less pronounced in the bladders from MMC-treated animals. This suggests that mechanosensory activity may rely less on TRPV1-positive afferents and more on non-TRPV1 mechanisms following MMC treatment, such as P2X, Piezo, ASIC, and TRPA1 channels [17, 23]. Recent work in a guinea pig model of cystitis suggested a role for Piezo1, but not ATP, in mechanotransduction in bladder afferents [23]. These results suggest a shift in afferent mechanisms of mechanotransduction rather than afferent populations. TRPV1 expression may not be altered, but its relative contribution is diluted because MMC pretreatment has amplified other mechanotransduction pathways. TRPV1 channels are also found on the urothelium and in control tissue TRPV1 channels contribute to mechanotransduction indirectly through the release of urothelial mediators or by interacting with mechanosensitive channels. MMC may disrupt this coupling, and even when TRPV1 is desensitised, the mechanosensory pathway remains active through alternative routes.

Another factor that may contribute to the afferent hyperactivity is an inflammatory response induced by MMC [24]. Intravesical MMC treatment induces a rise in bladder inflammation in patients and in urothelial cultures, with inflammatory markers elevated 24 h after MMC treatment [1, 11]. During local bladder inflammation, cytokines, histamine and oxidative stress can cause direct sensitization of bladder afferents [5, 9]. In further support for this hypothesis, the anti-inflammatory drug ibuprofen was found to reverse the voiding dysfunction following MMC treatment in our model [22]. Thus, local inflammatory responses induced by MMC may contribute to afferent hypersensitivity.

From a urological pharmacology perspective, these findings reveal targets for supportive co-therapies. Because the hypersensitivity is isolated to low-threshold physiological pathways, co-administering intravesical local anaesthetics or matching systemic therapies—such as beta3-adrenoceptor agonists or targeted P2 × 3 receptor antagonists—during or immediately following MMC instillation could selectively mute this premature afferent traffic. Mitigating these sensory side effects could improve patient compliance and optimise retention protocols for intravesical oncology regimens.

In conclusion, intravesical treatment with MMC results in altered bladder sensory nerve responses. Over the physiological range of pressures, afferent nerve activity was enhanced, and this may explain the storage symptom of urinary urgency and frequency encountered by patients after intravesical MMC treatment.

## Data Availability

The datasets generated during and/or analysed during the current study are available from the correspondingauthor on reasonable request.

## References

[1] Arends TJ, Falke J, Lammers RJ et al (2015) Urinary cytokines in patients treated with intravesical mitomycin-C with and without hyperthermia. World J Urol 33(10):1411–1417. 10.1007/s00345-014-1458-3 25491674

[2] Chess-Williams R, Chapple CR, Yamanishi T, Yasuda K, Sellers DJ (2001) The minor population of M3-receptors mediate contraction of human detrusor muscle in vitro. J Auton Pharmacol 21(5–6):243–248. 10.1046/j.1365-2680.2001.00231.x 12123469

[3] Daly D, Rong W, Chess-Williams R, Chapple C, Grundy D (2007) Bladder afferent sensitivity in wild-type and TRPV1 knockout mice. J Physiol 583(Pt 2):663–674. 10.1113/jphysiol.2007.139147 PMC2277033 17627983

[4] Daly DM, Chess-Williams R, Chapple C, Grundy D (2010) The inhibitory role of acetylcholine and muscarinic receptors in bladder afferent activity. Eur Urol 58(1):22–28. 10.1016/j.eururo.2009.12.030. (**Discussion 31– 2**) 20071074

[5] Dray A (1995) Inflammatory mediators of pain. Br J Anaesth 75(2):125–131. 10.1093/bja/75.2.125 7577246

[6] Filson CP, Montgomery JS, Dailey SM et al (2014) Complications associated with single-dose, perioperative mitomycin-C for patients undergoing bladder tumor resection. Urol Oncol 32(1):40e1–40e8. 10.1016/j.urolonc.2013.04.006 PMC4128494 23787296

[7] Forrest SL, Osborne PB, Keast JR (2013) Characterization of bladder sensory neurons in the context of myelination, receptors for pain modulators, and acute responses to bladder inflammation. Front Neurosci 7:206. 10.3389/fnins.2013.00206 PMC3819567 24223534

[8] Grundy L, Daly DM, Chapple C, Grundy D, Chess-Williams R (2018) TRPV1 enhances the afferent response to P2X receptor activation in the mouse urinary bladder. Sci Rep 8(1):197. 10.1038/s41598-017-18136-w PMC5760578 29317663

[9] Grundy L, Caldwell A, Garcia Caraballo S et al (2020) Histamine induces peripheral and central hypersensitivity to bladder distension via the histamine H(1) receptor and TRPV1. Am J Physiol Ren Physiol 318(2):F298–F314. 10.1152/ajprenal.00435.2019 31790304

[10] Hou JC, Landas S, Wang CY, Shapiro O (2011) Instillation of mitomycin C after transurethral resection of bladder cancer impairs wound healing: an animal model. Anticancer Res 31(3):929–932 21498715

[11] Kang SH, Chess-Williams R, Anoopkumar-Dukie S, McDermott C (2015) Recovery of urothelial mediator release but prolonged elevations in interleukin-8 and nitric oxide secretion following mitomycin C treatment. Naunyn Schmiedebergs Arch Pharmacol 388(7):781–791. 10.1007/s00210-015-1092-7 25644373

[12] Koch GE, Smelser WW, Chang SS (2021) Side effects of intravesical BCG and chemotherapy for bladder cancer: what they are and how to manage them. Urology 149:11–20. 10.1016/j.urology.2020.10.039 33181123

[13] Luckenbaugh AN, Marks RM, Miller DC, Weizer AZ, Stoffel JT, Montgomery JS (2017) A management algorithm for mitomycin C induced cystitis. Bladder Cancer 3(2):133–138. 10.3233/BLC-160089 PMC5409048 28516158

[14] Michielsen D, Amy JJ, Coomans D, Storme G, Wyndaele JJ (2005) Mitomycin C and epirubicin: functional bladder damage in rats after repeat intravesical instillations. J Urol 173(6):2166–2170. 10.1097/01.ju.0000158123.33273.f9 15879880

[15] Mills KA, West EJ, Grundy L et al (2020) Hypersensitivity of bladder low threshold, wide dynamic range, afferent fibres following treatment with the chemotherapeutic drugs cyclophosphamide and ifosfamide. Arch Toxicol 94(8):2785–2797. 10.1007/s00204-020-02773-8 32444959

[16] Mills KA, West EG, Sellers DJ, Chess-Williams R, McDermott C (2021) Psychological stress induced bladder overactivity in female mice is associated with enhanced afferent nerve activity. Sci Rep 11(1):17508. 10.1038/s41598-021-97053-5 PMC8410840 34471159

[17] Rong W, Spyer KM, Burnstock G (2002) Activation and sensitisation of low and high threshold afferent fibres mediated by P2X receptors in the mouse urinary bladder. J Physiol 541(Pt 2):591–600. 10.1113/jphysiol.2001.013469 PMC2290323 12042363

[18] Sellers DJ, Yamanishi T, Chapple CR, Couldwell C, Yasuda K, Chess-Williams R (2000) M3 muscarinic receptors but not M2 mediate contraction of the porcine detrusor muscle in vitro. J Auton Pharmacol 20(3):171–176. 10.1046/j.1365-2680.2000.00181.x 11193006

[19] Sharma H, Manning SK, Stevens NE et al (2025) Acute urinary tract infection elicits bladder afferent hypersensitivity. Brain Behav Immun Health 44:100944. 10.1016/j.bbih.2025.100944 PMC11788684 39901923

[20] Thrasher JB, Crawford ED (1992) Complications of intravesical chemotherapy. Urol Clin North Am 19(3):529–539 1636237

[21] Vlaskovska M, Kasakov L, Rong W et al (2001) P2X3 knock-out mice reveal a major sensory role for urothelially released ATP. J Neurosci 21(15):5670–5677. 10.1523/JNEUROSCI.21-15-05670.2001 PMC6762653 11466438

[22] West EG, Lang R, Sellers D, Chess-Williams R, McDermott C (2018) Ibuprofen decreases spontaneous activity and enhances nerve-evoked contractions to minimize mitomycin C-induced bladder dysfunction. J Pharmacol Exp Ther 366(2):282–290. 10.1124/jpet.118.248989 29784662

[23] Yew WP, Hibberd T, Spencer NJ, Zagorodnyuk V (2024) Piezo1, but not ATP, is required for mechanotransduction by bladder mucosal afferents in cystitis. Auton Neurosci 256:103231. 10.1016/j.autneu.2024.103231 39627069

[24] Yoshimura N, de Groat WC (1999) Increased excitability of afferent neurons innervating rat urinary bladder after chronic bladder inflammation. J Neurosci 19(11):4644–4653. 10.1523/JNEUROSCI.19-11-04644.1999 PMC6782608 10341262

[25] Yoshimura N, Oguchi T, Yokoyama H et al (2014) Bladder afferent hyperexcitability in bladder pain syndrome/interstitial cystitis. Int J Urol 21(0 1):18–25. 10.1111/iju.12308 PMC4089034 24807488

[26] Yu Y, de Groat WC (2010) Effects of stimulation of muscarinic receptors on bladder afferent nerves in the in vitro bladder-pelvic afferent nerve preparation of the rat. Brain Res 1361:43–53. 10.1016/j.brainres.2010.09.018 PMC3034278 20840844

